# Laparoscopic Organopexy with Non-mesh Genital (LONG) Suspension: A Novel Uterine Preservation Procedure for the Treatment of Apical Prolapse

**DOI:** 10.1038/s41598-018-23285-7

**Published:** 2018-03-20

**Authors:** Cheng-Yu Long, Chiu-Lin Wang, Chin-Ru Ker, Yung-Shun Juan, Eing-Mei Tsai, Kun-Ling Lin

**Affiliations:** 1 Department of Obstetrics and Gynecology, Kaohsiung Medical University Hospital, Kaohsiung Medical University, Kaohsiung, Taiwan; 2 Department of Obstetrics and Gynecology, Kaohsiung Municipal Hsiao-Kang Hospital, Kaohsiung Medical University, Kaohsiung, Taiwan; 3 Graduate Institute of Medicine, College of Medicine, Kaohsiung Medical University, Kaohsiung, Taiwan; 4 Department of Urology, Kaohsiung Medical University Hospital, Kaohsiung Medical University, Kaohsiung, Taiwan

## Abstract

To assess whether our novel uterus-sparing procedure- laparoscopic organopexy with non-mesh genital(LONG) suspension is an effective, safe, and timesaving surgery for the treatment of apical prolapse. Forty consecutive women with main uterine prolapse stage II or greater defined by the POP quantification(POP-Q) staging system were referred for LONG procedures at our hospitals. Clinical evaluations before and 6 months after surgery included pelvic examination, urodynamic study, and a personal interview to evaluate urinary and sexual symptoms with overactive bladder symptom score(OABSS), the short forms of Urogenital Distress Inventory(UDI-6) and Incontinence Impact Questionnaire(IIQ-7), and the Female Sexual Function Index(FSFI). After follow-up time of 12 to 30 months, anatomical cure rate was 85%(34/40), and the success rates for apical, anterior, and posterior vaginal prolapse were 95%(38/40), 85%(34/40), and 97.5%(39/40), respectively. Six recurrences of anterior vaginal wall all suffered from significant cystocele (stage3; Ba>+1) preoperatively. The average operative time was 73.1 ± 30.8 minutes. One bladder injury occurred and was recognized during surgery. The dyspareunia domain and total FSFI scores of the twelve sexually-active premenopausal women improved postoperatively in a significant manner (P < 0.05). The results of our study suggest that LONG suspension is an effective and safe uterus-sparing surgery for the treatment of apical prolapse.

## Introduction

Pelvic organ prolapse (POP) is a chronic illness in women that has received growing attention among gynecologists worldwide because of the aging population and people’s pursuit of life quality. In search for a safe and effective treatment strategy, various surgical techniques have been evolved, such as vaginal hysterectomy and/or anterior-posterior colporrhaphy, sacrospinous fixation, laparotomy or laparoscopic sacrocolpopexy, and transvaginal mesh (TVM) implantation. Studies and debates have been devoted to compare the objective and subjective surgical outcomes of these approaches, and some consensuses were reached^1^.

Firstly, sacral colpopexy was considered the gold standard procedure in treating apical prolapse^1^. With the advent of minimal invasive methods, laparoscopic or robot-assisted laparoscopic sacrocolpopexy has comparable anatomic correction and patient satisfaction rates, with the advantages of less blood loss, shorter hospital stay and reduced medical cost^2–4^. However, anterior vaginal prolapse recurrence is common because the anterior compartment fixation becomes compromised with the posterior compartment fixation at the sacrum. Longer operating time and learning curve restricted its popularity as well. Secondly, synthetic graft implantation, once considered a breakthrough innovation in repairing POP in experienced hands, was criticized for the insufficient evidence that support its safety^5^. The enthusiasm for mesh-augmented POP repairs further declined after USFDA publicly issued a warning in 2011 for the same reason^5^. Maher *et al*. later added to this issue by showing the superiority of laparoscopic sacrocolpopexy to total vaginal mesh procedure in reduced blood loss, reduced reoperation rates, no mesh-related complications and greater patient satisfaction in a randomized controlled trial^6^. Thirdly, an increasing number of women are choosing not to have hysterectomy for reasons of personal identity, perceived body image, or childbearing potential apart from the common contraindications such as cervical dysplasia, uterine pathology, and postmenopausal uterine bleeding, etc^7,8^. Furthermore, hysterectomy and uterine preservation seems to have comparable anatomical outcomes after TVM surgery^9^.

Taking the above-mentioned concerns altogether, we invented a novel uterus-sparing procedure- Laparoscopic Organopexy with Non-mesh Genital (LONG) Suspension for the treatment of apical prolapse. The procedure is designed to create ventral uterine suspension to the transversalis fascia underneath rectus abdominis without use of mesh. Reviewing the literature, most uterine suspension procedures are performed either vaginally or laparoscopically with synthetic meshes, and non-mesh and ventral uterine suspension has never been reported. The aim of our study was to assess whether LONG suspensionis an effective, safe, and timesaving surgery. In addition, surgical complications and functional outcomes were also evaluated.

## Results

The demographic data of the enrolled 40 women are shown in Table 1. The ages of all participants ranged from 37 to 78 years, with an average of 58.3 years; parity ranged from 0 to 4, with a mean of 2.1. Twenty-six (65%) patients were postmenopausal and only one (2.5%) was under hormone therapy. None had previous prolapse surgery. Either anterior or posterior colporrhaphy with the LONG suspension were performed in two (5%) women; another single patient (2.5%) had combined myomectomy and LONG operation. Concomitant mid-urethral slings were done in four (10%) women, and all of them were free of stress urinary incontinence (SUI) postoperatively. None of the twenty-two preoperative continent women developed de novo SUI.

**Table 1:** Demographic data (n = 40) are given as mean ± standard deviation or n (%).

Parameters	Mean ± Std or N (%)
Mean age (years)	58.3 ± 13.0
Mean parity	2.1 ± 1.2
Mean body mass index (kg/m^2^)	22.6 ± 2.4
Menopause	26 (65)
Current hormone therapy	1 (2.5)
Diabetes mellitus	2 (5)
Hypertension	16 (40)
Baseline apical POP stage 2	21 (52.5)
with anterior POP stage 2	8 (25)
with anterior POP stage 3	2 (5)
with posterior POP stage 2	1 (2.5)
apical POP stage 3	19 (47.5)
with anterior POP stage 3	4 (10)
Concomitant procedures in this study	
Anterior colporrhaphy	1 (2.5)
Posterior colporrhaphy	1 (2.5)
Myomectomy	1 (2.5)
Midurethral sling	4 (10)
Follow-up (months)	2–30

BMI, body mass index; POP, pelvic organ prolapse; SUI, stress urinary incontinence; Sx, surgery.

**Figure 1:**
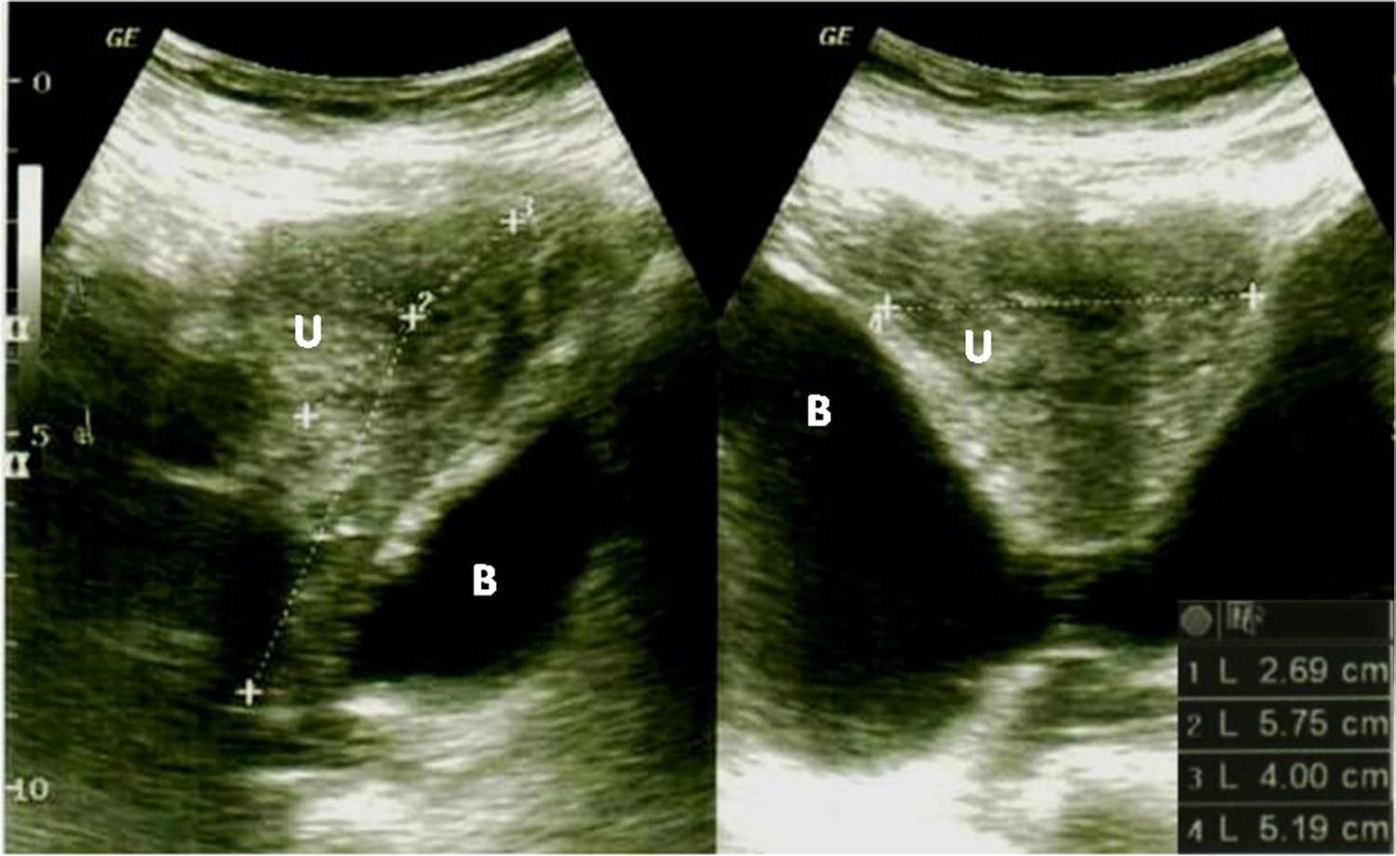
Good attachment of uterus to the abdominal wall can be observed on ultrasound after LONG suspension.

After follow-up time of 12 to 30 months, there was a significant improvement at points Aa, Ba, C, Ap, Bp, and total vaginal length (P < 0.01; Wilcoxon signed rank test). Anatomical cure rate was 85% (34/40), and the success rates for apical, anterior, and posterior vaginal prolapse were 95% (38/40), 85% (34/40), and 97.5% (39/40), respectively (Table 2). Good attachment of uterus to the abdominal wall can be observed on ultrasound in women with successful outcomes (Fig. 1). Six recurrences of anterior vaginal compartment occurred at 2–6 months after LONG suspension, and they all suffered from significant cystocele (stage 3; Ba>+1) preoperatively; one underwent vaginal hysterectomy plus sacrospinous ligament fixation; two had TVM surgery; and the remaining three did not desire additional surgery due to asymptomatic POP.

**Table 2:** Pelvic organ prolapse quantification (POP-Q) values before and after surgery. Data are given as median (range) or N (%).

POP-Q parameters (cm)	Pre-OP (n = 77)	Post-OP (n = 77)	P values*
Aa	−1 (−3~2)	−2 (−3~−2)	<0.001^#^
Ba	1 (−3~3)	−2 (−3~−2)	<0.001^#^
C	1 (−2~3)	−7 (−4~−9)	<0.001^#^
Ap	−2 (−3~0)	−3 (−3~−2)	0.005^#^
Bp	1 (−3~2)	−3 (−5~0)	0.003^#^
Tvl	9 (7~10.5)	10 (9~10)	0.002^#^

Pre-OP, preoperative; Post-OP, postoperative; Tvl, total vaginal length. *Wilcoxon signed rank test. ^#^Statistical significance.

The prevalences of urinary symptoms, including SUI, urge incontinence, and incomplete bladder emptying, were found to be significantly lower 6 months after LONG surgery (P < 0.01; McNemar’s test) (Table 3). Other symptoms, such as urinary frequency, urinary hesitancy, and nocturia improved, but did not show significant difference postoperatively. In addition, postoperative scores of OABSS, UDI-6, and IIQ-7 decreased significantly (P < 0.05; Paired *t*-test) (Table 3). Twenty women (20/40; 50%) were diagnosed as OAB by OABSS records and the OABSS scores improved in 15 (75%) women following surgical correction. None experienced de novo OAB symptoms.

**Table 3:** Urinary symptoms and quality of life questionnaires before and 6 months after surgery. Data are given as N (%).

Symptoms	Pre-OP (n = 40)	Post-OP (n = 40)	P values
Daytime frequency	14 (35)	7 (17.5)	0.07*
Stress urinary incontinence	18 (45)	6 (15)	<0.001*^∞^
Urge urinary incontinence	16 (40)	6 (15)	0.012 *^∞^
Feeling of incomplete emptying	15 (37.5)	2 (5)	0.002 ^+ ∞^
Hesitancy	7 (17.5)	2 (5)	0.063^+^
Nocturia	22 (55)	17 (42.5)	0.45*
OABSS	3.6 ± 1.7	1.5 ± 0.5	<0.01^‡∞^
UDI-6	57.5 ± 38.0	42.0 ± 25.5	0.02^‡∞^
IIQ-7	32.9 ± 18.2	21.2 ± 11.7	0.038^‡∞^

OABSS: Overactive Bladder Symptom Score; UDI-6: the short forms of Urogenital Distress Inventory; IIQ-7: the Incontinence Impact Questionnaire. *McNemar’s test, ^+^Fisher’s exact test, ^‡^Paired t-test ^∞^Statistical significance.

The residual urine decreased in a significant manner postoperatively (P < 0.05; Table 4). However, the rate of DO and other urodynamic parameters, including maximum flow rate, maximum cystometric capacity, maximum urethral closure pressure and urethral closure area revealed that analogous parameters were not significantly different following the LONG procedures (P > 0.05; Paired *t*-test) (Table 4). The average operative time was 73.1 ± 30.8 minutes. One bladder injury occurred and was recognized during surgery, with laparoscopic closure of bladder rupture being carried out immediately. Postoperative day1 VAS score was 2.4 ± 1.3. Urinary tract infection occurred in five women (5/40; 12.5%) (Table 5). All complications according to the Clavien-Dindo classification are summarized in Table 6.

**Table 4:** Urodynamic changes before and 6 months after surgery. Data are given as n (%) or mean ± standard deviation.

Parameters (cm)	Pre-OP (n = 40)	Post-OP (n = 40)	P values
Qmax (ml/s)	18.0 ± 5.1	17.8 ± 6.4	0.91
RU (ml)	25.6 ± 13.3	13.6 ± 6.8	0.036^‡∞^
DO	9 (22.5)	4 (10)	0.18*^‡^
FS (ml)	164.6 ± 73.9	154.5 ± 64.3	0.23^‡^
MCC (ml)	399.6 ± 39.4	393.4 ± 93.4	0.67^‡^
Pdet (cmH2O)	29.5 + 19.9	28.0 ± 17.9	0.52^‡^
FUL (mm)	27.4 ± 3.4	26.4. ± 3.8	0.16^‡^
MUCP (cmH2O)	56.3 ± 26.2	60.1 ± 31.2	0.23^‡^

DO: destrusor overactivity; Qmax: maximum flow rate; RU: residual urine; FS: first sensation to void; MCC: maximum cystometric capacity; Pdet: detrusor pressure at peak flow; FUL: functional urethral length; MUCP: maximum urethral closure pressure. *Fisher’s exact test; ^‡^Paired t-test. ^∞^Statistical significance.

**Table 5:** Intra-operative and postoperative complications. Data are given as n (%).

Parameters	N = 40
Intraoperative complications	
Operative time (minutes)	73.1 ± 30.8
Bladder injury	1 (2.5)
Rectal injury	0
Blood transfusion	0
Conversion to laparotomy	0
Postoperative complications	
Post-op day 1 VAS score	2.4 ± 1.3
Urinary tract infection	5 (12.5)
Voiding dysfunction	0
Pelvic hematoma	0

**Table 6:** Complications of LONG surgery according to the Clavien-Dindo classification grade.

Clavien-Dindo classification	Postoperative UTI	Postoperative VD	Bladder injury
Grade I			
Grade II	5/40 (12.5%)	0	
Grade III			1/40 (2.5%)
Grade IV			
Total	5/40 (12.5)	0	1/40 (2.5%)

UTI: urinary tract infection; VD: voiding dysfunction.

When evaluating the changes in sexual function, only 12 sexually-active premenopausal women were included in this study. The domains of FSFI, including sexual desire, sexual arousal, lubrication, orgasm, and satisfaction, were not significantly different following LONG suspension (P > 0.05). However, the dyspareunia domain and total scores improved postoperatively in a significant manner (P < 0.05) (Table 7).

**Table 7:** Changes in scores of Female Sexual Function Index before and 6 months after LONG surgery. Data are given as mean ± standard deviation.

Domains	Pre-OP (n = 12)	Post-OP (n = 12)	P values
Sexual desire	3.2 ± 0.8	3.4 ± 0.3	0.31
Sexual arousal	3.9 ± 0.6	4.0 ± 0.9	0.34
Lubrication	5.7 ± 2.1	5.6 ± 0.3	0.28
Orgasm	5.6 ± 0.4	5.6 ± 0.6	0.17
Satisfaction	5.5 ± 0.8	5.5 ± 0.7	0.17
Dyspareunia	3.7 ± 1.4	5.4 ± 0.6	0.009^+^
Total scores	27.5 ± 2.5	29.4 ± 2.2	0.037^+^

Pre-OP, preoperatively; Post-OP, postoperatively; *Paired t-test; ^+^Statistical significance.

## Discussion

Among abdominal uterine preservation surgeries, laparoscopic sacrohysteropexy has remained a gold standard for the treatment of apical prolapse^1^. However, the procedure has some limitations such as access into the pararectal and perisacral spaces, the area of retroperitoneal nerve plexuses, and non-physiologic axis of the vagina associated with a possible risk of vascular and recurrent complications. This technique is suitable for experienced operators who have excellent orientation in pelvic anatomy with the focus on retroperitoneal and rectovaginal space, although longer learning curve and operative time has restricted its popularity as well.

Recently, TVM surgery has gained popularity over the last decade due to the excellent short-term cure rate, especially in the anterior compartment^10,11^. Moreover, hysteropexy and hysterectomy seems to have comparable surgical outcomes after TVM surgery^9^. However, the United States Food and Drug Administration (FDA) announced a public health notification regarding “serious complications associated with transvaginal placement of surgical mesh in repair of POP and stress urinary incontinence (SUI)” on 2011 July^5^. Indeed, the FDA warning has raised further caution when selecting the type of mesh kits.

The mean age of 58 years for all subjects in this study was the same as women undergoing laparoscopic sacrohysteropexy in a recent report^12^, but younger than the age of 63 years in our previous study of TVM^10^. This implies similar age groups of subjects among laparoscopic hysteropexy procedures. Recently, hysteropexy using the mesh procedure has gained more and more popularity, irrespective abdominally, laparoscopically, or vaginally^1–6^. Laparoscopic uterine suspension without mesh has never been reported except for the Gilliam round-ligament uterine ventro-suspension procedure^13^. A review study concluded that Laparoscopic uterine ventro-suspension using round ligaments has a very limited role, with a success rate less than 50%^14^. It is apparent that elongation of round ligament by uterine weight causes a higher recurrence of uterine prolapse. LONG suspension yields a favorable result by modifying the anchorage of uterine body to the transversalis fascia in spite of ventral suspension, reaching an apical success rate of 95%.

Theoretically, uterine ventral suspension causes an upward traction of anterior vaginal wall, helping to correct the anterior compartment prolapse as well. However, LONG surgery created an anatomical cure rate of 85% in the anterior compartment, slightly lower than a recent study showing 9% recurrent anterior prolapse following laparoscopic sacral hysteropexy^12^. This maybe owing to the fact that only one anterior colporrhaphy was performed together in these six women with anterior vaginal recurrences. Six anterior vaginal recurrences occurred from women all with cystocele stage 3 (Ba>+1) preoperatively. Therefore, LONG surgery seems to be particularly suitable in women with main uterine prolapse. Concomitant anterior and/or posterior colporrhaphy might be necessary in women with significant cystocele and/or rectocele (over stage 2), although it looked like mild cystocele at the end of LONG procedure.

One bladder injury occurred in the second case, during the dissection of fat and transversalis fascia. The average operative time for the first 10 cases was 87.5 minutes and 58.5 minutes for cases 11–40, indicating short learning curve existed in this procedure. Compared with TVM, comparable outcomes were also observed in both surgeries. In theory, the less the amount of mesh placed, the lower the rate of extrusion occurrence. Despite the type I Lite mesh(lower molecular weight) used in TVM surgery, the vaginal extrusion rate still reached 6.6% in a recent study^12^. There were only minimal to moderate and/or absence of mesh-related complications in the LONG suspension procedures.

Over one-third of women undergoing LONG surgery will have multi-compartmental prolapse. It would therefore be impractical to identify a population with only uterine prolapse. Dysfunctional voiding in itself may be multi-factorial and is considered to be mainly related to anterior and/or apical vaginal prolapse. As expected, postoperative improvement of incomplete emptying symptom was observed in our patients, as evidenced by significant decrease of residual urine on urodynamics. The cure of SUI was mainly due to the combination of mid-urethral sling with the LONG surgery in several women. Originally, we worried that postoperative higher rate of OAB due to posterior bladder compression of the uterus might occur; on the contrary, we found the irritating symptoms and OABSS scores improved following LONG suspension, and none experienced de novo OAB symptoms. The rate of women with preoperative OAB was 50%, identical to the figure of a previous study^15^.

We once reported that premenopausal women might experience higher rate of deteriorated dyspareunia following TVM surgery than postmenopausal women^16^. Additionally, women undergoing total mesh surgery had greater sexual impairment in comparison to women with anterior mesh alone^17^. The weak points of TVM remain vaginal scarring and sensory impairment caused by mesh implantation. It is worth emphasizing that the dyspareunia domain and total scores of FSFI improved significantly following LONG suspension, similar to the results of laparoscopic sacrocolpopexy^18^. The Taiwan translation of the FSFI has been validated for linguistic accuracy^19^. This finding was also associated with the non-mesh nature in this novel procedure.

Some may question the possibility of intestinal obstruction because the small bowel might be incaserated into the space between uterus and urinary bladder. We are sure that no women met this condition after the follow-up time of 12–30 months. This could be partly explained by no visible space between uterus and bladder from ultrasound image (Fig. 1). Another concern might arise about the feasibility of subsequent bladder-related surgery or hysterectomy when uterine pathology appears. We believe the above-mentioned surgeries can be carried out easily due to only 3 stitches being done between the uterus and abdominal fascia.

The results of our study suggest that LONG suspension is an effective and safe uterus-sparing surgery for the treatment of apical prolapse. It takes advantages over laparoscopic sacrocolpopexy and TVM with time-saving performance and better functional outcome respectively. A flaw of this study was the absence of women with stage 4 uterine prolapse. This was due to the fact that women with severe uterine prolapse were scheduled for vaginal hysterectomy during this study period due to cervical elongation, hypertrophic uterus, history of endometrial pathology, and postmenopausal bleeding. Of course, more case numbers and longer follow-up time are necessary to confirm the durability and safety of this novel procedure.

## Methods

From April 2014 through October 2016, 48 consecutive women with main uterine prolapse stage II or greater as defined by the POP quantification (POP-Q) staging system^20^, were referred for LONG procedure at our hospitals. We excluded women with cervical elongation, hypertrophic uterus, prior TVM recurrence, history of cervical dysplasia or endometrial pathology, and postmenopausal bleeding in the past 12 months. Concomitant anti-incontinence sling operations were performed in women with current or occult urodynamic stress incontinence (USI), unless they did not desire a concomitant surgery. All patients gave their written informed consent before surgery. These mid-urethral sling procedures included MiniArc (AMS, Inc., Minnetonka, MN, USA), and TVT-O (Gynecare TVT-Obturator System, Ethicon, Inc., Somerville, NJ). Eight women were excluded due to various reasons, including incomplete medical records (n = 5), and loss of follow-up (n = 3). Finally, the remaining 40 women were included for analysis in this cohort study.

Clinical evaluations before and 6 months after surgery included pelvic examination using the POP-Q system, multichannel urodynamic study, transabdominal ultrasound, and a personal interview to evaluate overactive bladder symptom score (OABSS)^21^, the short forms of Urogenital Distress Inventory (UDI-6), the Incontinence Impact Questionnaire (IIQ-7)^22^, the Female Sexual Function Index (FSFI) questionnaire^23^, and urinary symptoms with the standardized questionnaire taking into account the 2002 ICS definitions^24^. Women were asked to fill out the VAS (visual analogue scale) scores during the postoperative day1 rounds. Urodynamic studies, including non-instrumented uroflowmetry, filling and voiding cystometry, and urethral pressure profilometry, were performed by the recommendations by the International Continence Society^25^ with a multichannel urodynamic monitor (MMS; UD2000, Enschede, The Netherlands). USI was diagnosed by involuntary urine leakage with cough in the absence of detrusor contractions during filling cystometry. The diagnosis of occult USI was made by the occurrence of urine loss during the reduction of POP. Any uninhibited detrusor contraction during filling cystometry was deemed positive for idiopathic detrusor overactivity (DO).

### Operative technique

**Figure 2:**
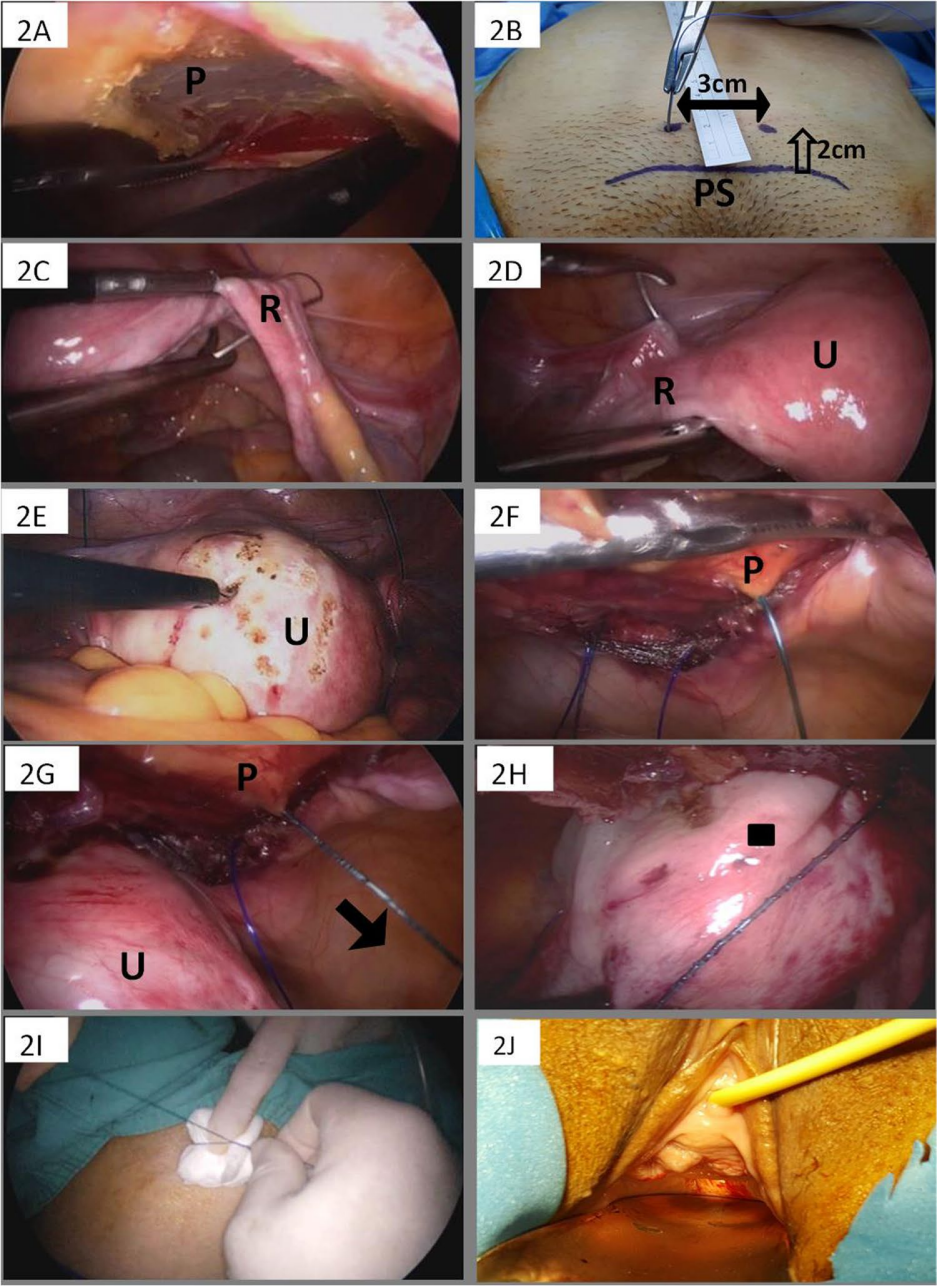
The procedures of laparoscopic organopexy with non-mesh genital (LONG) suspension.

The procedure is described as follows: Under laparoscopy, the peritoneum lining the abdominal wall above the uterus is dissected carefully until reaching the transversalis fascia underneath rectus abdominis (Fig. 2A). A Prolene 1.0 (Prolene, Ethicon, Inc., Somerville, NJ, USA) is inserted at the point 2 cm above the pubic symphysis, leaving at least 3 cm of the suture material extracorporeally (Fig. 2B) for a fixation tie in the final step. An anchorage is then created using the Prolene 1.0 to surround the uterus, starting from penetrating the broad ligament on the right side (Fig. 2C), bypassing the posterior aspect of the uterus to the broad ligament on the opposite side (Fig. 2D) and finally exiting the Prolene 1.0 from the abdominal wall about 3 cm horizontally away from the entry site. An abrasion wound over the anterior surface of the uterus (Fig. 2E) is made to augment adhesion effect between the uterus and the abdominal wall. Fixation of the uterus to the transversalis fascia is then performed with 3 stitches of a V-loc suture (Covidien, Mansfield, MA, USA) (Fig. 2F,G). Tisseel (Baxter; Deerfield, Italy) could be applied to promote hemostasis among surrounding tissues (Fig. 2H), completing the intra-abdominal steps. Finally, a fixation tie with the Prolene 1.0 (used at the beginning of the operation) is made over a folded gauze as a cushion outside the body (Fig. 2I). The tie and the gauze cushion (prevention of skin collpase) are left in place for 2 weeks, when wound healing is thought to be completed and adhesion between the uterus and abdominal wall stabilized. The tie and gauze cushion can be removed easily in the outpatient settings. Pelvic examination showed good outcomes after surgery (Fig. 2J), and Foley catheterization for one day.

As follow-up, postoperative outpatient visits were at 1, 2, 3, 6, and 12 months and then semiannually beyond one year. Pelvic examination was performed routinely in every visit of clinics. Recurrence was defined as the most dependent portion of POP stage II or greater. The Clavien-Dindo grading was used for the classification of the complications of LONG procedure^26^. A statistical analysis was performed using Paired t-test, or Wilcoxon signed rank test for continuous variables, and McNemar’s test for categorical variables. A difference was considered statistically significant when p < 0.05. The study protocols were approved by the Institutional Review Board of Kaohsiung Medical University Hospital, by which relevant guidelines and regulations were followed accordingly.

We assessed the power of tests for differentiating the surgical outcomes of LONG procedure, and power analysis showed that around 36–40 women in this study would have a power of 80%. Although some comparisons, such as DO rates, could not reach sufficient power due to the limited numbers, we utilized multiple parameters of POP-Q system to evaluate the postoperative change. We found that our subjects over 32 women, there would be a power of over 85% for discrimination.

### Data Availability Statement

The datasets analyzed during the current study are available from the corresponding author on reasonable request.

### Ethical Approval and Informed Consent

Ethics approval by the Institutional Review Board of Kaohsiung Medical University Hospital had been obtained for data analysis.
